# Combined transfection of Bcl-2 siRNA and miR-15a oligonucleotides enhanced methotrexate-induced apoptosis in Raji cells

**DOI:** 10.7497/j.issn.2095-3941.2013.01.003

**Published:** 2013-03

**Authors:** Li Ding, Xiao-Mao Hu, Hong Wu, Ge-Xiu Liu, Yang-Jun Gao, Dong-Mei He, Yuan Zhang

**Affiliations:** Institute of Hematology, Jinan University, Guangzhou 510632, China

**Keywords:** B-cell lymphoma 2 (Bcl-2), small interfering RNA, oligonucleotide, methotrexate, Raji cell, miR-15a, apoptosis

## Abstract

**Objective:**

B-cell lymphoma 2 (Bcl-2) is an important member of the Bcl-2 family of proteins that regulate the induction of apoptosis. This study aims to investigate whether Bcl-2 small interfering RNA (siRNA) combined with miR-15a oligonucleotides (ODN) could enhance methotrexate (MTX)-induced apoptosis in Raji cells.

**Methods:**

Chemically synthesized miR-15a ODN and Bcl-2 siRNA were transfected in Raji cells by using a HiPerFect Transfection Reagent and then combined with MTX. Expression levels of Bcl-2 protein were detected by Western blot. Cell proliferation was determined by CCK8 assay. The rate of cell apoptosis was determined by Annexin V/PI double staining. The morphology of apoptotic cells was observed by Hoechst-33 258 staining.

**Results:**

After the cells were transfected with miR-15a ODN combined with Bcl-2 siRNA, Bcl-2 protein levels were evidently decreased. CCK8 assay showed that cell proliferation was significantly decreased and was significantly lower in miR-15a ODN combined with Bcl-2 siRNA plus MTX group than in miR-15a ODN with methotrexate group, Bcl-2 siRNA with MTX group, and single MTX group (*P*<0.05). Hoechst 33258 staining revealed numerous apoptotic cells. AnnexinV/PI double staining showed that the apoptotic rates were (13.13±1.60)%, (34.47±2.96)%, (32.87±3.48)%, and (45.47±2.16)% in MTX, Bcl-2 siRNA plus MTX, miR-15a ODN plus MTX, and miR-15a ODN combined with Bcl-2 siRNA plus MTX groups, respectively. Among these groups, the apoptotic rate of miR-15a ODN combined with Bcl-2 siRNA plus MTX group was the highest; this apoptotic rate was also significantly different from that of miR-15a ODN or Bcl-2 siRNA plus MTX (*P*<0.05).

**Conclusions:**

Bcl-2 siRNA combined with miR-15a ODN could enhance MTX-induced apoptosis in Raji cells. Bcl-2 siRNA and miR-15a combined with MTX may be a useful approach to improve the treatment effects on lymphoma.

## Introduction

MicroRNAs (miRNAs) are short non-coding RNAs with 19 to 24 nucleotides that can regulate gene expression by an imperfect base pairing with complementary sequences located mainly in 3’ untranslated regions of target mRNAs^1^. miRNAs mainly induce translational suppression in eukaryotic cells and may function as tumor suppressor genes or potential oncogenes during the initiation and progression of cancer^1^^-^^4^. miR-15a is located at chromosome 13q14, which is frequently deleted in B-cell chronic lymphocytic leukemia^5^^,^^6^. Studies have shown that deregulated expression of miR-15a induces apoptosis by targeting the antiapoptotic protein B-cell lymphoma 2 (Bcl-2)^7^^,^^8^. Bcl-2 expression has been observed in a majority of human cancer specimens and cell lines. Bcl-2 protein regulates the mitochondria-mediated apoptosis pathway. High levels of Bcl-2 expression are associated with resistance to chemotherapeutic agents in numerous tumor types. Thus, a drug that can reduce the levels of this protein can promote apoptosis and can be considered a promising therapeutic agent^9^^,^^10^.

Small interfering RNAs (siRNAs) that target Bcl-2 mRNA can effectively induce apoptosis of lymphoma and leukemia cells^11^^-^^13^. We found that miR-15a oligonucleotides (ODN) and siRNA targeting against Bcl-2 mRNA alone can induce apoptosis in Raji cells *in vitro*^13^^,^^14^. Gao *et al*.^15^ showed that miR-15a/16-1 may function as a tumor suppressor to regulate leukemic cell proliferation potentially by downregulating WT1 oncogene. Combined treatment of methotrexate (MTX) and irradiation significantly induces apoptosis and growth inhibition in nasal NK/T-cell lymphoma cells^16^. However, whether Bcl-2 siRNA combined with miR-15a ODN could enhance MTX-induced apoptosis of lymphoma Raji cells remains unknown. This study aims to investigate the effect of combined transfection of Bcl-2 siRNA and miR-15a ODN on MTX-induced apoptosis of Raji cells. Therefore, our study suggested that the combined transfection of Bcl-2 siRNA and miR-15a ODN plus MTX could provide an efficacious therapeutic approach to treat lymphoma that expresses Bcl-2.

## Materials and methods

### Reagents

Bcl-2-specific (Bcl-2-195) siRNA, negative control siRNA (corresponding non-silencing negative control siRNA) and miR-15a ODN (a scrambled ODN) were synthesized by Shanghai Genechem Biotechnology Co., Ltd (Shanghai, China). RPMI-1640 and newborn calf serum were purchased from Gibco (NY, USA). MTX was purchased from Shanxi Powerdone Pharmaceutics Co., Ltd (Shanxi, China).

### Cell culture and transfection

Raji cell line was purchased from the Shanghai Cell Bank. The cells were cultured in RPMI medium supplemented with 10% heat-inactivated fetal calf serum at 37 °C under 5% CO_2_ in a humidified incubator. Raji cells in the exponential phase were grown for 24 h and then transfected using HiPerFect Reagent (Qiagen, Valencia, CA) according to the manufacturer’s protocols. In addition to mock transfected control (mock) with HiPerFect Reagents, the cells were transfected with negative siRNA and scrambled ODN. The total concentration of siRNA or ODN applied in every case was held constant at 100 nM. For combined treatments with Bcl-2 siRNA and miR-15a, the ratio of Bcl-2 siRNA to miR-15a was maintained at 1^:^1.

### Western blot assay

The cells were washed with PBS (10 mM, pH 7.4), incubated in 200 mL of cell lysis buffer (50 mmol/L TrisHCl, 5 mmol/L EDTA, 25% sucrose, 0.6 mL of 100 mg/mL phenylmethylsulphonyl fluoride, and 2.4 mL of 2-mexca-ptoethanol) in an ice bath for 30 min, and centrifuged at 13,000×*g* for 45 min at 4 °C. The protein of the cell lysate was determined by Bio-Rad assay according to the manufacturer’s protocol. Whole cell extracts equivalent to 100 µg of the total protein were separated by 8% sodium dodecyl sulfate polyacrylamide gel electrophoresis and electrotransferred to nitrocellulose membranes (Gibco BRL, USA). The blot was placed in a blocking buffer (10% non-fat dry milk, 1% Tween-20; in 20 mM Tris-buffered saline, pH 7.5) for 1 h at room temperature and incubated with appropriate anti-human primary antibody (rabbit anti-human Bcl-2/IgG, 1:300; rabbit anti-human GAPDH/IgG, 1:1000; Santa Cruz) in blocking buffer overnight at 4 °C. Blots were incubated with anti-rabbit horseradish peroxidase-conjugated secondary antibody (1^:^1000; Santa Cruz) for 1 h and detected by chemiluminescence using ECL Hyperfilm.

### Assay of cell viability

We performed CCK8 assay to determine cellular proliferation and activity quantitatively. This assay was performed after Raji cells were transfected with Bcl-2 siRNA and miR-15a combined with MTX (45 µM) at 24, 48, and 72 h. The cells were washed, counted, and seeded at a density of 4×10^5^ cells/mL in each well in 96-well plates. After 6 h, Bcl-2 siRNA and miR-15a combined with MTX were added to the cells. At 24, 48, and 72 h after transfection, CCK8 solution was added 4 h before incubation was completed. Cell viability was determined with a spectrophotometer at an absorbance of 450 nm. The inhibition rates of cell growth were calculated according to the following equation: inhibition rate (%) = (1-mean absorbance of treatment group/mean absorbance of untreated group) × 100%.

### Assays of cell apoptosis

Transfected Raji cells were harvested after treatment. Morphology was determined with Hoechst 33258 after these cells were incubated for 48 h. The cells were washed with PBS thrice and then stained with 10 µL of Hoechst 33258 nuclear dye for 10 min at 37 °C. After the cells were washed again with PBS thrice, images were obtained by fluorescence microscopy (Leica, Germany). Apoptosis detection kit (Annexin V binding) was used according to the manufacturer’s instructions. In brief, the cells were centrifuged, washed with cold PBS, and resuspended in 500 µL of binding buffer. Fluoresce isothiocyanate conjugated Annexin V (10 µL) and propidium iodide (PI, 10 µL) were added to each sample, and the mixture was incubated at 4 °C in the dark for 5 min. The cells were immediately subjected to FACS analysis (BD FAC-S Calibur, USA). The percentages of early and late apoptotic cells in each group were determined.

### Statistical analysis

Results are shown as mean±SD. Statistical comparisons were made by ANOVA. Differences were considered significant at a real alpha of 0.05. Statistical analyses were performed with SPSS 13.0 [Norman H. Nie, C. Hadlai (Tex) Hull and Dale H. Bent, Chicago, USA].

## Results

### Bcl-2 siRNA combined with miR-15a suppressed Bcl-2 protein expression in Raji cells

**Figure 1:**
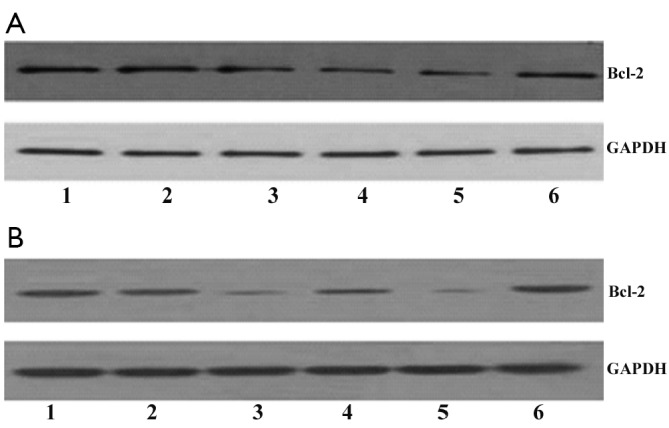
Effect of Bcl-2 siRNA combined with miR-15a on Bcl-2 protein expression levels in Raji cells. Bcl-2 protein levels were determined by Western blot analysis at 24 h (A) and 48 h (B). GAPDH was used as a loading control. Lanes 1 to 6: untreated cells, negative siRNA, Bcl-2 siRNA, miR-15a ODN, miR-15a ODN plus Bcl-2siRNA, and scrambled ODN, respectively.

Bcl-2 protein levels were assayed by Western blot. The results indicated that Bcl-2 protein expression decreased at 24 and 48 h in cells transfected with Bcl-2 siRNA or miR-15a compared with either negative control siRNA or untreated cells (**Figure 1**). No difference was observed in Bcl-2 levels among negative control siRNA group, mock-transfected cells, and untransfected cells. Bcl-2 protein expression was more effectively reduced by combining Bcl-2 siRNA and miR-15a compared with either Bcl-2 siRNA treatment or miR-15a treatment alone.

### Bcl-2 siRNA and miR-15a combined with MTX affected the growth of Raji cells

**Figure 2:**
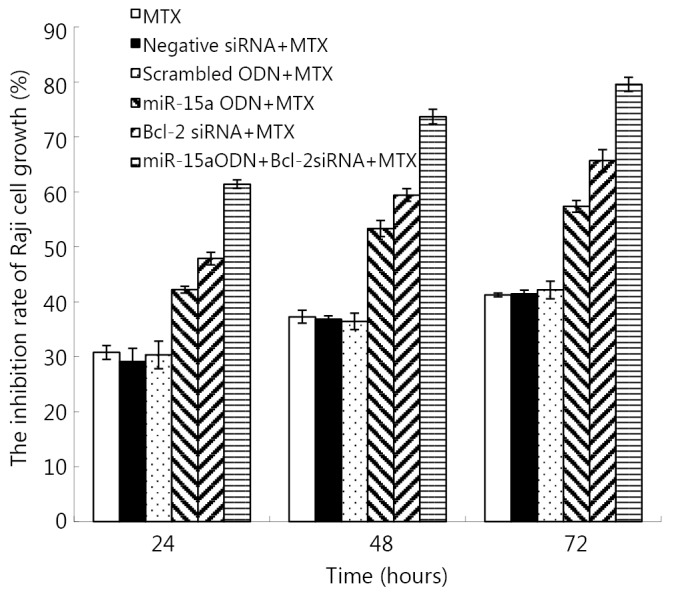
Inhibition rate of Raji cell growth by CCK8 assay after these cells were subjected to combined transfection with Bcl-2 siRNA and miR-15a ODN plus MTX.

CCK8 assays were performed to investigate the effects of transfection of Bcl-2 siRNA and miR-15a alone or in combination with MTX on the growth of Raji cells. A summary of experiments measuring cell death at 24, 48, and 72 h is shown in **Figure 2**. Although Bcl-2 siRNA and miR-15a could inhibit the growth of Raji cells, the viability of the cells was more effectively reduced by combining MTX in a time-dependent manner (*P*<0.05).

### Bcl-2 siRNA combined with miR-15a enhanced MTX-induced apoptosis in Raji cells

**Figure 3:**
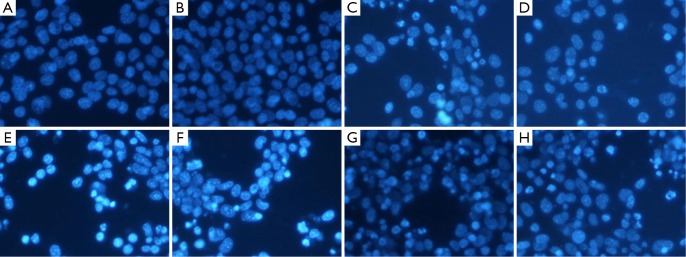
Effect of combined transfection with miR-15a and Bcl-2 siRNA plus MTX on the morphology of Raji cells at 48 h. Nuclear morphology of cells stained with Hoechst-33 258 was analyzed by fluorescence microscopy (×200) at 48 h after transfection. Data are representative microscopic pictures. Untreated cells (A); negative siRNA (B); MTX (C); negative siRNA + MTX (D); scrambled ODN + MTX (E); miR-15a ODN + MTX (F); Bcl-2 siRNA + MTX (G); and miR-15a ODN + Bcl-2 siRNA + MTX (H).

**Figure 4:**
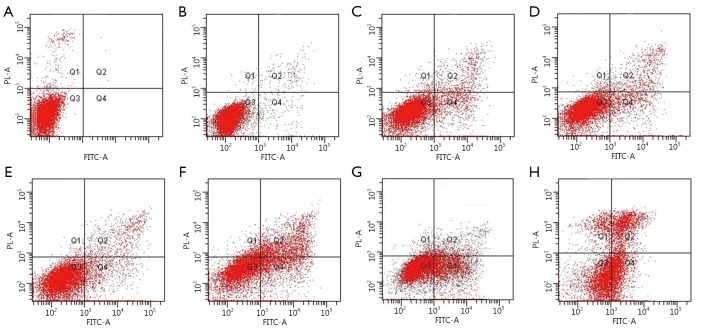
Rate of apoptosis induced by combined transfection with miR-15a and Bcl-2 siRNA plus MTX in Raji cells at 48 h. Apoptosis was analyzed by AnnexinV-FITC/PI staining at 48 h after treatment. Data are representative pictures. Untreated cells (A); negative siRNA (B); MTX (C); negative siRNA + MTX (D); scrambled ODN + MTX (E); miR-15a ODN + MTX (F); Bcl-2 siRNA + MTX (G); and miR-15a ODN + Bcl-2 siRNA + MTX (H)

Two methods were used to assess apoptosis: (1) Hoechst staining and (2) Annexin V-FITC/PI double staining by FACS analysis. **Figure 3** shows that the cells in the control group exhibited normal nuclear morphology. At 48 h after transfection with Bcl-2 siRNA and miR-15a ODN combined with MTX in Raji cells, significant nuclear condensation and morphological changes such as chromatin condensation and fragmentation were observed. AnnexinV-FITC and PI double staining with FACS were also used to detect cell apoptosis. **Figure 4** shows that AnnexinV- FITC/PI double positive cells increased at 48 h after transfection with Bcl-2 siRNA and miR-15a combined with MTX in Raji cells. The apoptotic rate in Bcl-2 siRNA and miR-15a ODN combined with MTX group was (45.47±2.16)%. This apoptotic rate was significantly different compared with miR-15a ODN plus MTX group, Bcl-2 siRNA plus MTX group, or MTX alone group (*P*<0.05). The apoptotic rates in these three groups were (32.87±3.48)%, (34.47±2.96)%, and (13.13±1.60)%, respectively. These results also had a significant difference compared with the negative siRNA control plus MTX group [(13.33±0.72)%] and the scrambled ODN plus MTX group [(13.57±1.07)%].

## Discussion

MiRNAs are abnormally expressed in many human malignancies^1^^,^^4^^,^^17^. MiR-15a/16-1 cluster is frequently deleted and downregulated in B-CLL^5^^-^^7^. MiR-15b and miR-16 modulate the sensitivity of gastric cancer cells to some anticancer drugs by targeting Bcl-2^18^. Our previous study showed that miR-15a is significantly downregulated in human lymphoma Raji cells and that miR-15a overexpression suppresses the growth of Raji cells^14^. Our previous studies also showed that a reduction in Bcl-2 expression by siRNA can induce apoptosis in Raji cells^13^.

In the present study, the combined transfection of Bcl-2 siRNA and miR-15a was more effective in reducing Bcl-2 protein levels in Raji cells. Bcl-2 knockdown by siRNA and miR-15a ODN enhanced MTX, thereby inhibiting the growth of Raji cells. Apoptotic effect and reduction in the number of cells of the 3 combined treatments were significantly greater than those of treatment with MTX alone, Bcl-2 siRNA plus MTX, or miR-15a ODN plus MTX. This result may be accounted for an enhanced downregulation of Bcl-2 by Bcl-2 siRNA and miR-15a ODN. In our previous study, Bcl-2 siRNA treatment combined with miR-15a ODN decreases the proliferation and increases the number of apoptotic cells compared with Bcl-2 siRNA or miR-15a ODN transfection alone^19^.

A greater inhibition of tumor growth and viability possibly result from the inhibition of multiple physiological events mediating malignancy and tumor cell survival or a single critical pathway at multiple points to maximize the inhibition of this pathway^20^. For instance, combined treatment of miR-15a/16-1 and arsenic trioxide synergistically induces apoptosis in K562, NB4, U937, Raji cells, and primary leukemic cells from 5 cases of patients with AML^21^. Studies have also suggested that the combined treatment of two siRNAs elicits a significant increase in antiviral effects of RNAi against hepatitis C virus *in vitro*^22^.

Previous studies also reported that the downregulation of Bcl-2 expression by siRNA increases the effect of doxorubicin on a canine mammary gland tumor cell line^13^^,^^23^. Another study has shown that Bcl-2 siRNA-mediated gene silencing increases the sensitivity of human hepatoblastoma HepG2 cells to chemotherapeutic drugs, including 5-fluorouracil and 10-hydroxycamptothecin^24^.

The present study indicated that the combined treatment of Bcl-2 siRNA and miR-15a increased MTX-induced apoptosis in Raji cells. Our study also suggested that the combined treatment of Bcl-2 siRNA and miR-15a mimics plus MTX could be potentially used as a novel therapy for human lymphomas. However, further preclinical research is required.
